# Presenting Symptoms of Undiagnosed Autism Spectrum Disorder Among Young Boys and Girls in Community CAMHS Between 2018–2019

**DOI:** 10.1192/bjo.2022.482

**Published:** 2022-06-20

**Authors:** Kesegofetse Setlhare, Sobia Rafi

**Affiliations:** Black Country Healthcare NHS Foundation Trust, Wolverhampton, United Kingdom

## Abstract

**Aims:**

Though Autism Spectrum Disorder (ASD) is a common childhood neurodevelopmental disorders, the literature on presentation of undiagnosed ASD in Consultant Child and Adolescent Mental Health Services, (CAMHS) is scarce. The aim of the study was to look at symptoms at presentation among boys and girls in CAMHS, compare the symptom profile between the two genders, establish the main referral and assessment pathways and interventions employed after diagnosis

**Methods:**

This was a retrospective review of patients’ files referred to ASD Walsall CAHMS Clinic conducted in February 2021. A random sample size of 44 boys and girls equally distributed from the ASD database was selected randomly from the completed ASD assessment list, the equal distribution between genders was intentional. We looked at presenting symptoms reported on the referral letters, assessments in CAMHS, and interventions outlined from ASD outcome letters of all subjects with completed ASD assessment, in age groups 7–18 years.

**Results:**

Across genders, most patients presented in the teenage years with common age of presentation seen at ages 15 and 17, both at 15.9% and mean age being 13 years. Ninety-five percent of patients were in school at the time of referral. Only 4.5% of patient were referred through crisis and the rest through local GP. A variety of presenting symptoms were seen, with the majority of the patients presenting with social and communication difficulties (77.3%), under /overreaction to sensory stimuli (63.6%) and anxiety (61.4%). 9.1% of patients had a family history of ASD. 100% of assessments included ADOS, SALT and neurodevelopmental assessment. 77.3% of patients were referred to support groups like living with ASD parent support groups. Along with CAMHS, education (97.7%) was the main agency involved in the care of these patients. In 44.2% of patients, EHCP was requested or already in place. The in between gender comparison also showed that although most symptoms were similar in both groups, some such as self-harm were higher among girls (27.3%) as compared to boys (13.6%) as well as obsessional symptoms which were more common in boys (63.3%) as compared to girls (27.3%).

**Conclusion:**

Undiagnosed ASD presents with a wide variety of symptoms amongst boys and girls. Previous UK studies have shown an earlier presentation of ASD and which is contrary to our findings demonstrating a much later presentation. Therefore, we recommend referrers to be aware of the varied presentations and have a lower threshold for referral to secondary services to aid quicker ASD diagnosis and management.

